# Supplementary material for: A Disila[2]ferrocenophane with a Bridging 9,9′-Bi-9H-9-Silafluorene Moiety
Source: Molecules. 2025 Mar 18;30(6):1361. doi: 10.3390/molecules30061361 (PMC11944698; doi:10.3390/molecules30061361)
Supplement: Supplementary file 1 [file molecules-30-01361-s001.zip › molecules-3537647-supplementary.pdf]

Supporting Information for

A Disila[2]ferrocenophane with a Bridging 9,9′-Bi-9*H*-9-silafluorene Moiety

Shinnosuke Usuba,[1] Shogo Morisako,[2,3] Koichiro Masada,[3,4] Koh Sugamata,[3,4] and Takahiro Sasamori[3,4,*]

[1]Graduate School of Science and Technology, University of Tsukuba, 1-1-1 Tennoudai, Tsukuba, Ibaraki 305-8571, Japan,

[2]Sagami Chemical Research Institute, Hayakawa 2743-1, Ayase, Kanagawa 252-1193, Japan

[3]Department of Chemistry, Institute of Pure and Applied Sciences, University of Tsukuba, 1-1-1 Ten-noudai, Tsukuba, Ibaraki 305-8571, Japan

[4]Tsukuba Research Center for Energy Materials Sciences (TREMS), University of Tsukuba, 1-1-1 Ten-noudai, Tsukuba, Ibaraki 305-8571, Japan

E-mail: sasamori@chem.tsukuba.ac.jp

Tel, Fax: +81-29-853-4412

Contents

1. Spectra of all novel compounds S3
2. Cartesian coordinates of theoretically optimized structures S17

1. NMR spectra of all novel compounds

*9-Chloro-9-diethylamino-9-silafluorene (**3**)*

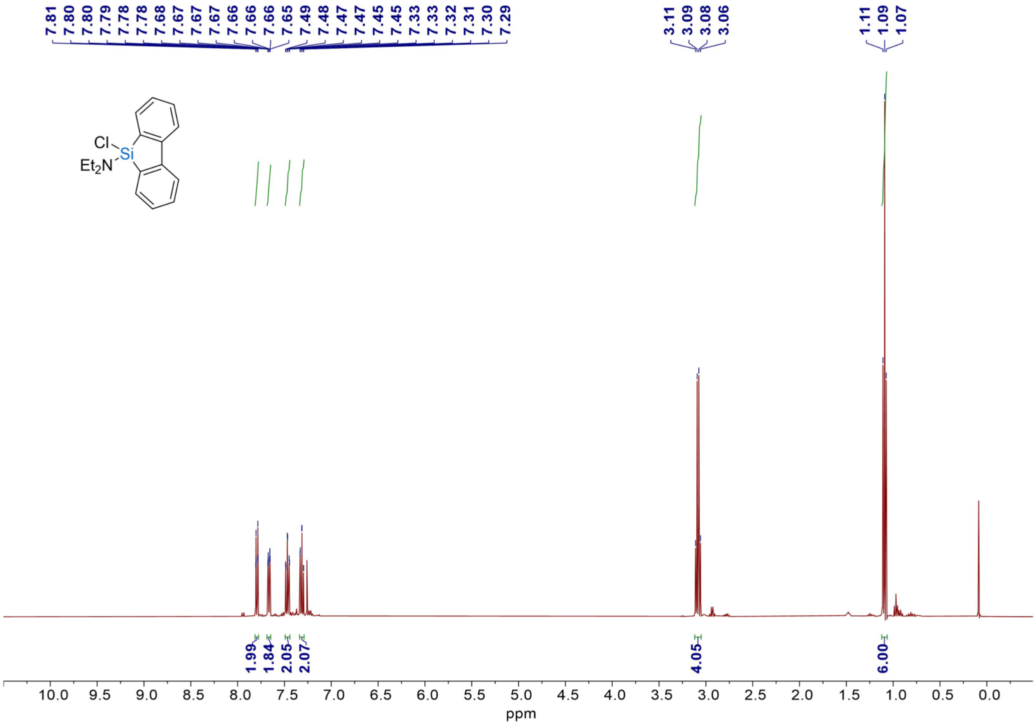

Figure S1. ^{1}H NMR spectrum of **3** ($CDCl_3$, 400 MHz).

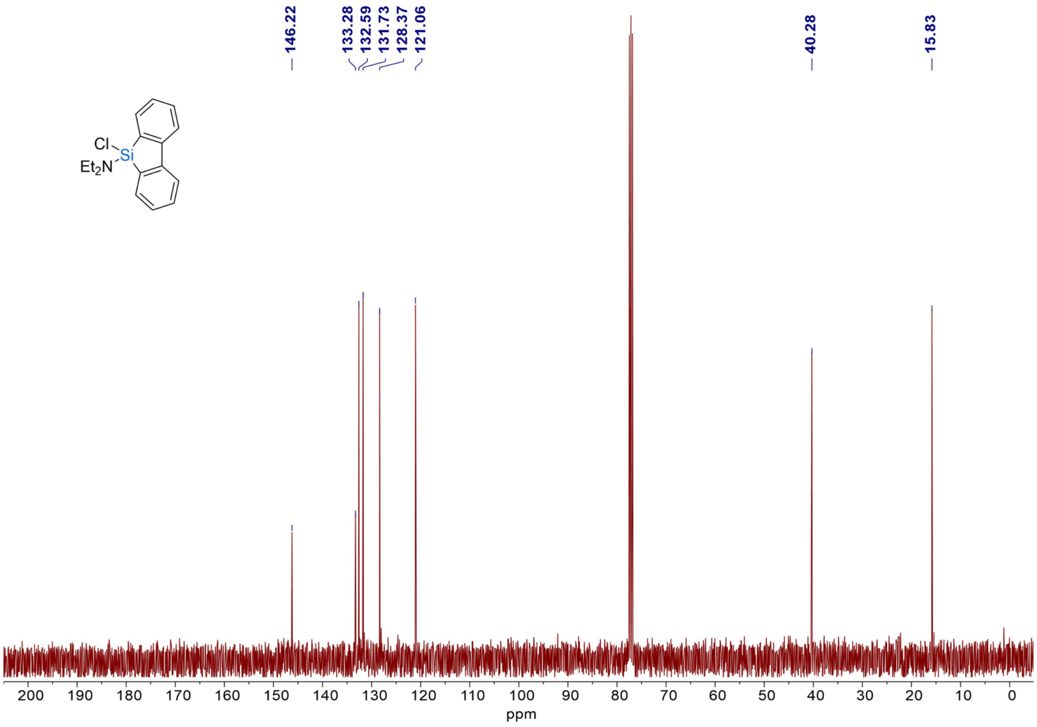

Figure S2. $^{13}C\{^{1}H\}$ NMR spectrum of **3** ($CDCl_3$, 101 MHz).

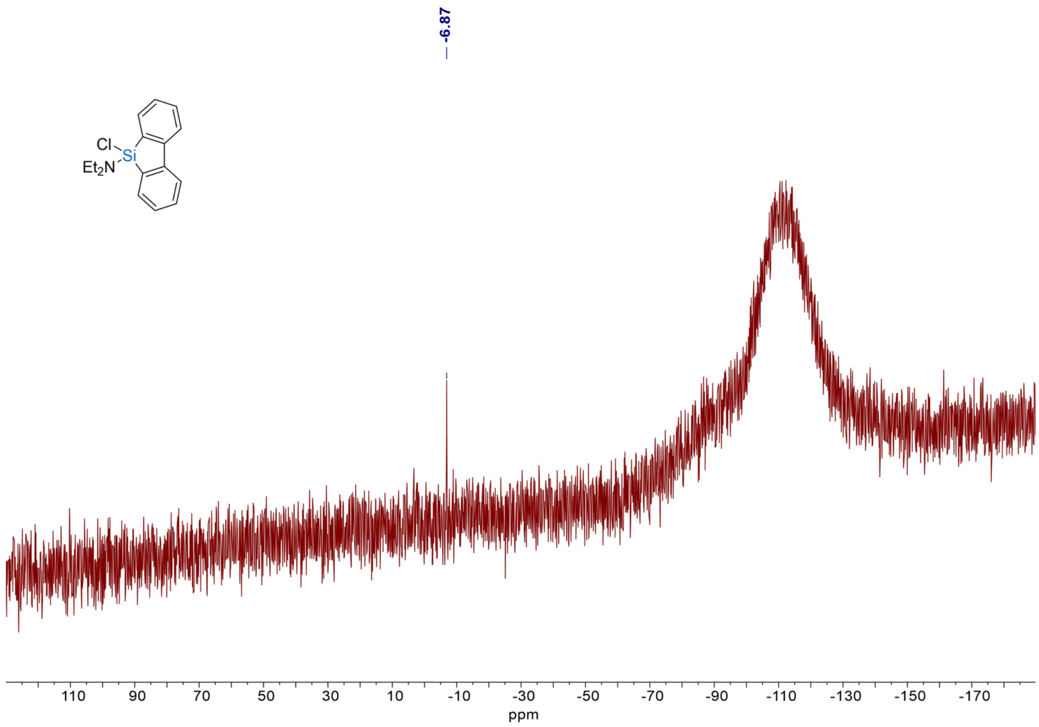

Figure S3. $^{29}Si\{^{1}H\}$ NMR spectrum of **3** (C_6D_6, 79.5 MHz).

*1,1'-Bis(9-diethylamino-9-silafluorenyl)ferrocene (**4**)*

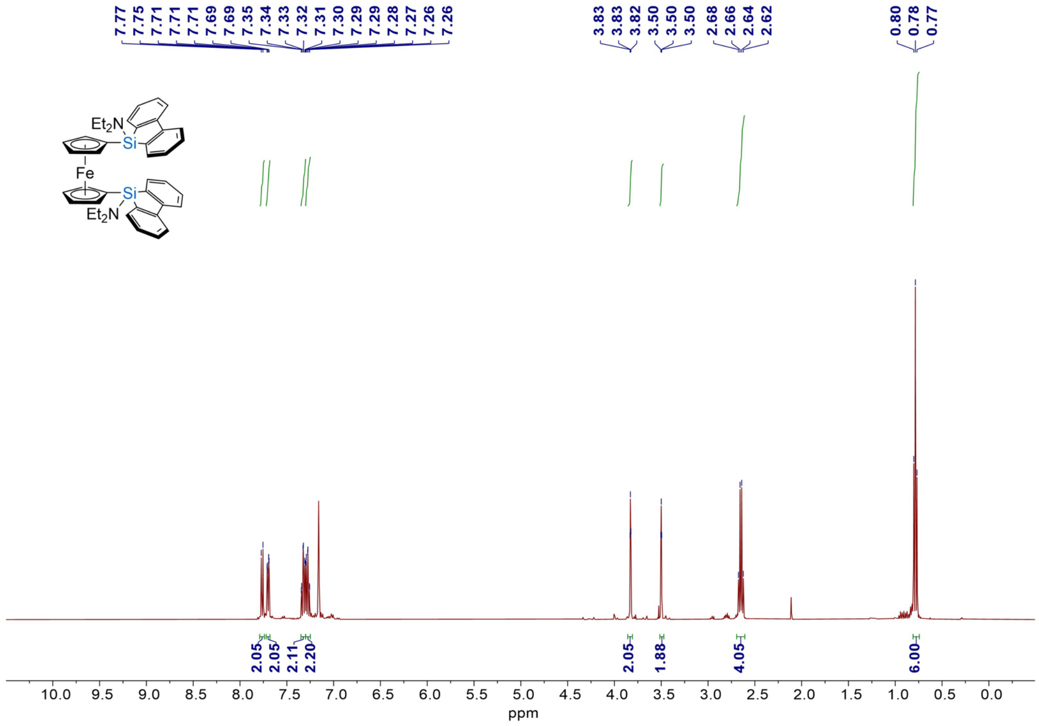

Figure S4. ^{1}H NMR spectrum of **4** (C_6D_6, 400 MHz).

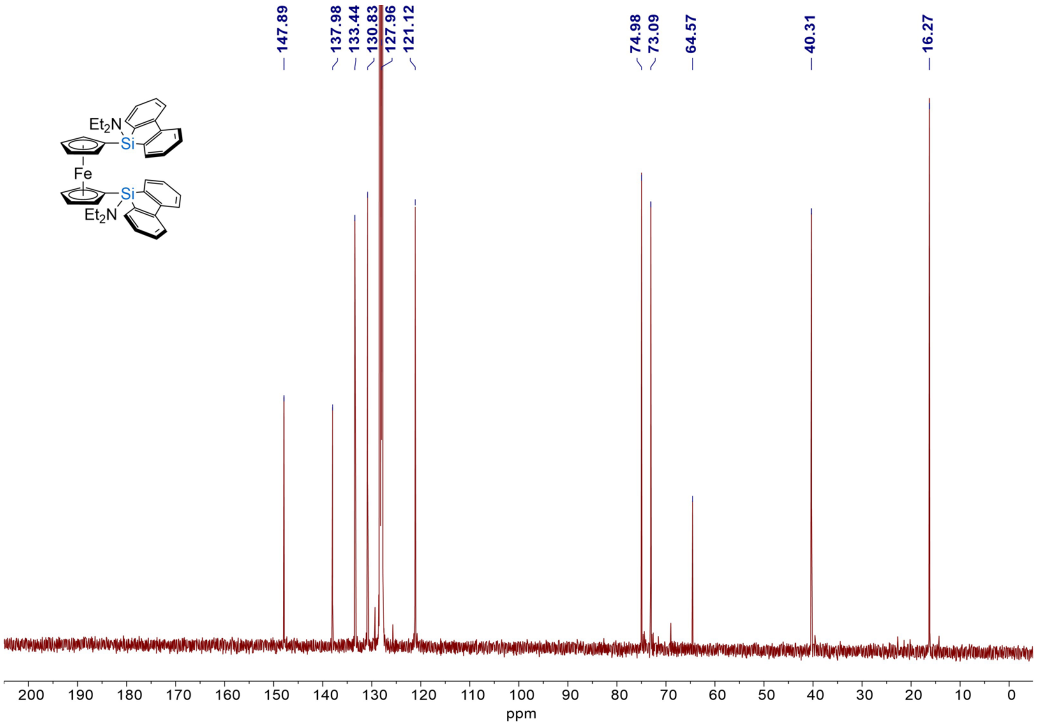

Figure S5. $^{13}C\{^1H\}$ NMR spectrum of **4** (C_6D_6, 101 MHz).

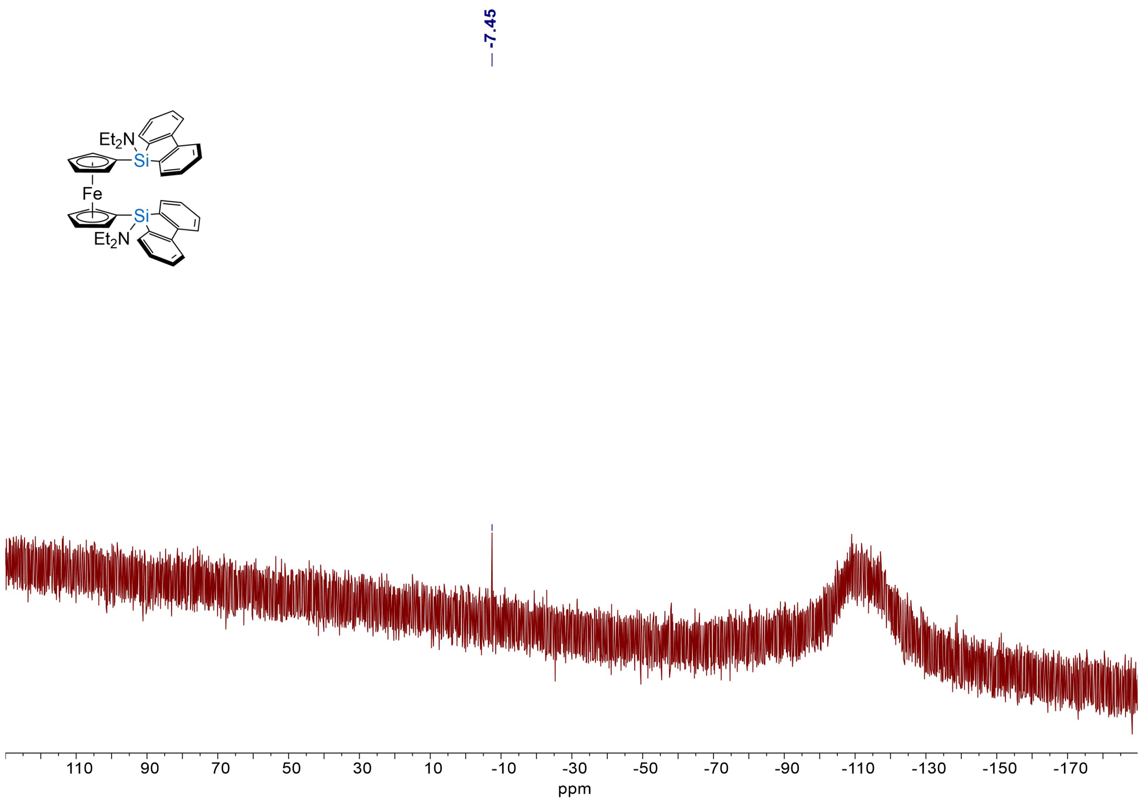

Figure S6. $^{29}Si\{^1H\}$ NMR spectrum of **4** (C_6D_6, 79.5 MHz).

*1,1'-Bis(9-chloro-9-silafluorenyl)ferrocene (**5**)*

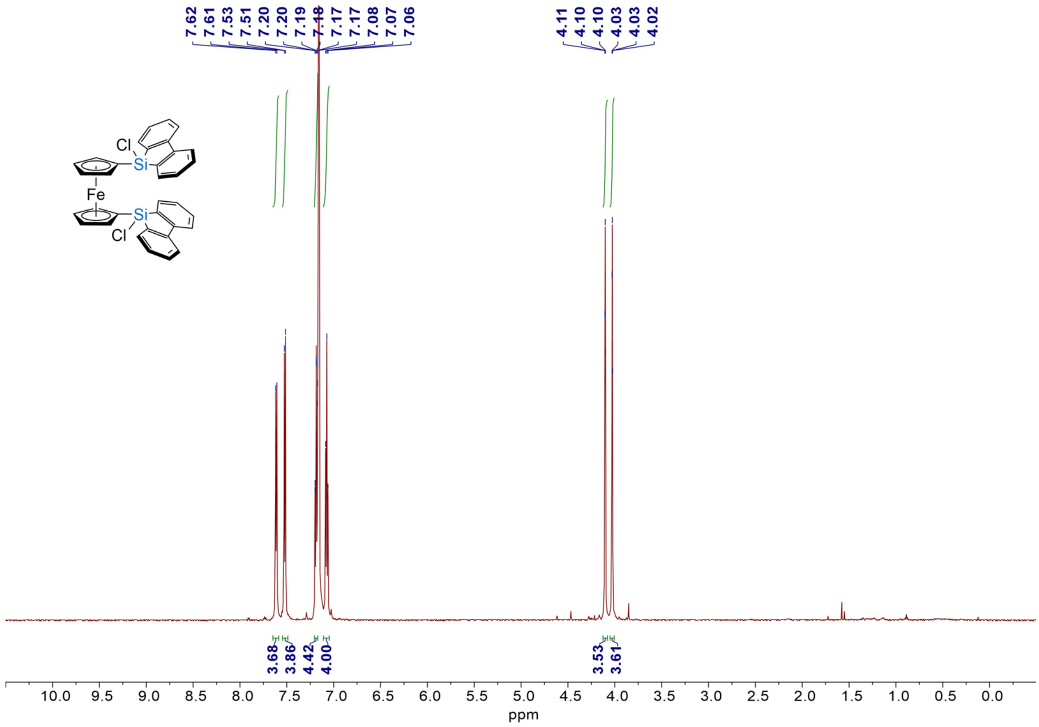

Figure S7. ^{1}H NMR spectrum of **5** (C_6D_6, 600 MHz).

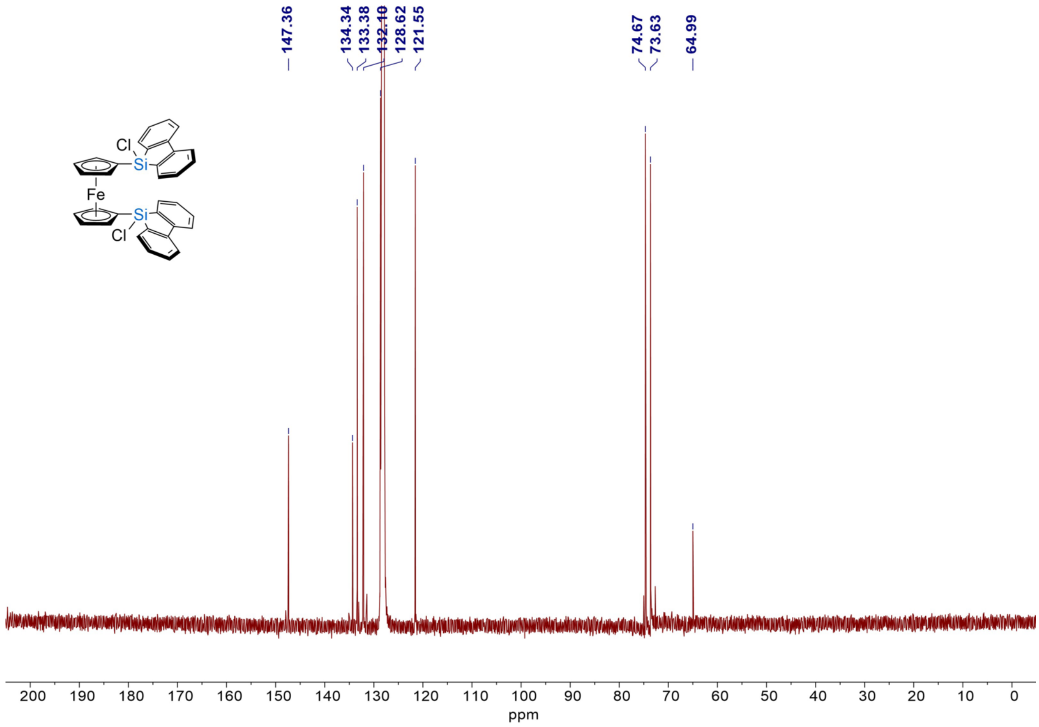

Figure S8. $^{13}C\{^{1}H\}$ NMR spectrum of **5** (C_6D_6, 151 MHz).

*Disila[2]ferrocenophane **1***

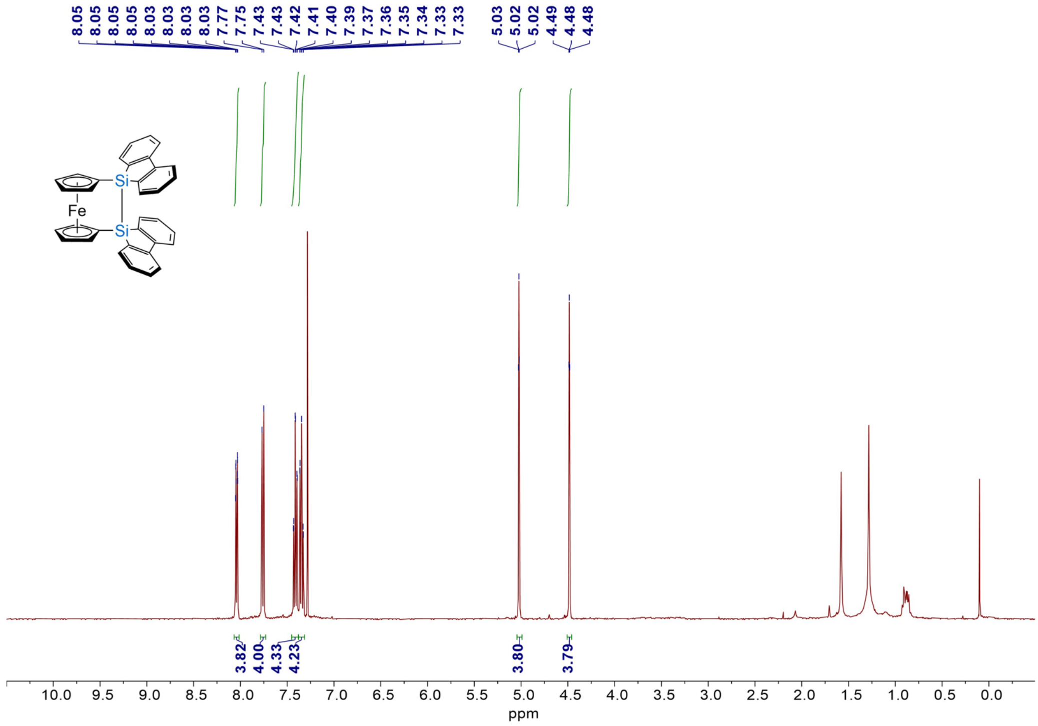

Figure S9. ^{1}H NMR spectrum of **1** (C_6D_6, 400 MHz).

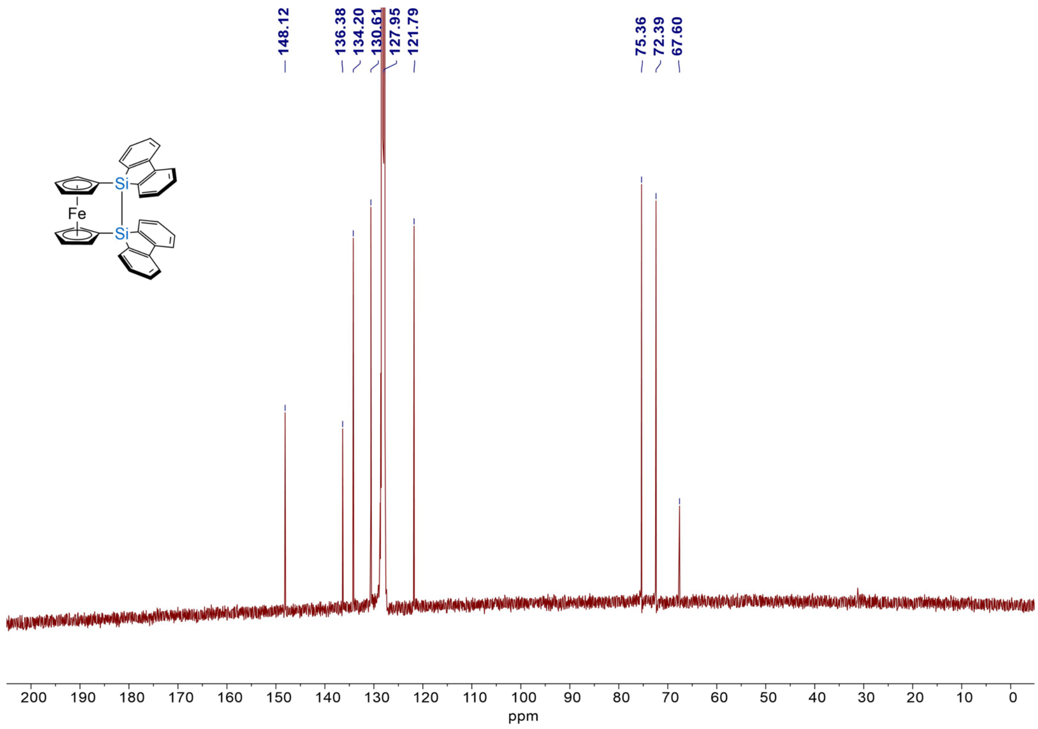

Figure S10. $^{13}C\{^{1}H\}$ NMR spectrum of **1** (C_6D_6, 101 MHz).

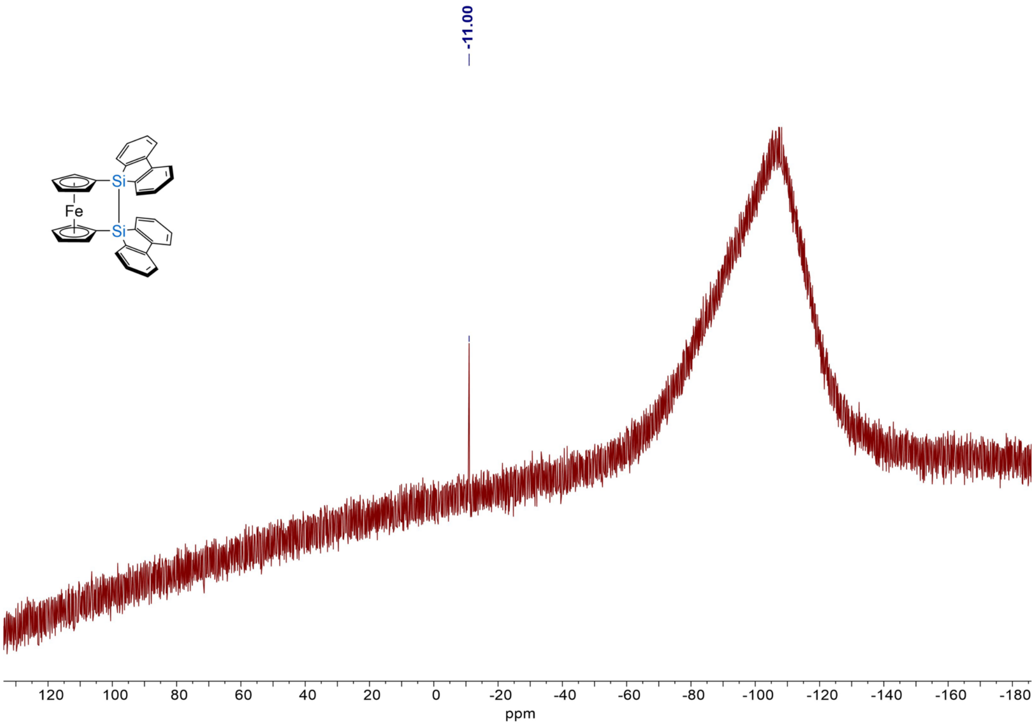

Figure S11. $^{29}Si\{^1H\}$ NMR spectrum of **1** (C_6D_6, 79.5 MHz).

*1,1'-Bis(9-trimethylsilyl-9-silafluorenyl)ferrocene (**6**)*

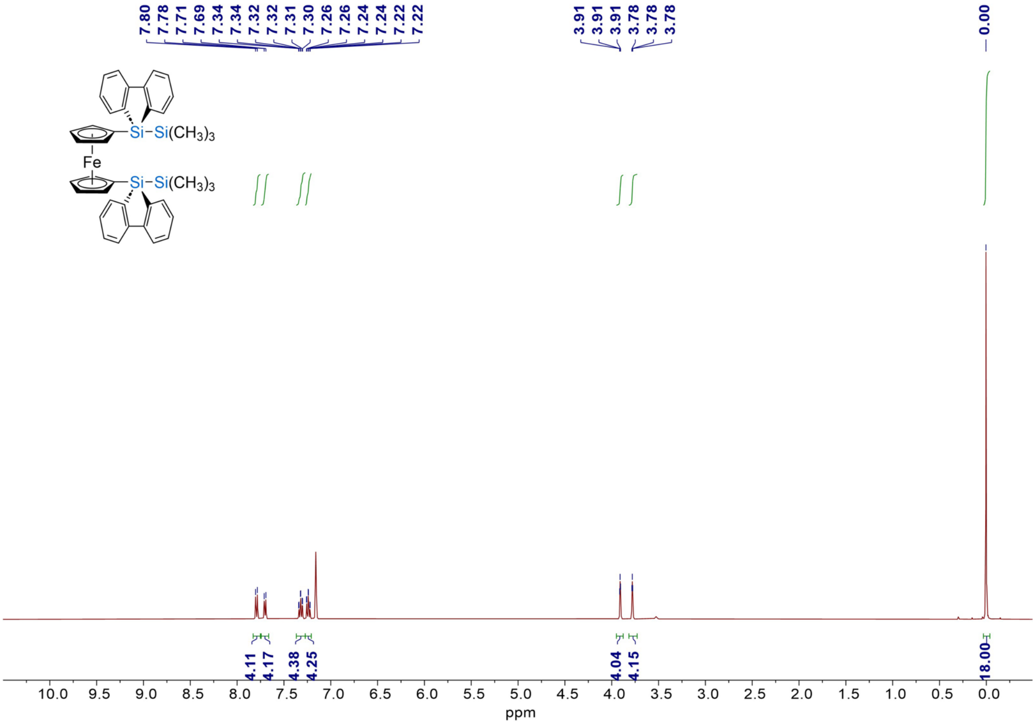

Figure S12. ^1H NMR spectrum of **6** (C_6D_6, 400 MHz).

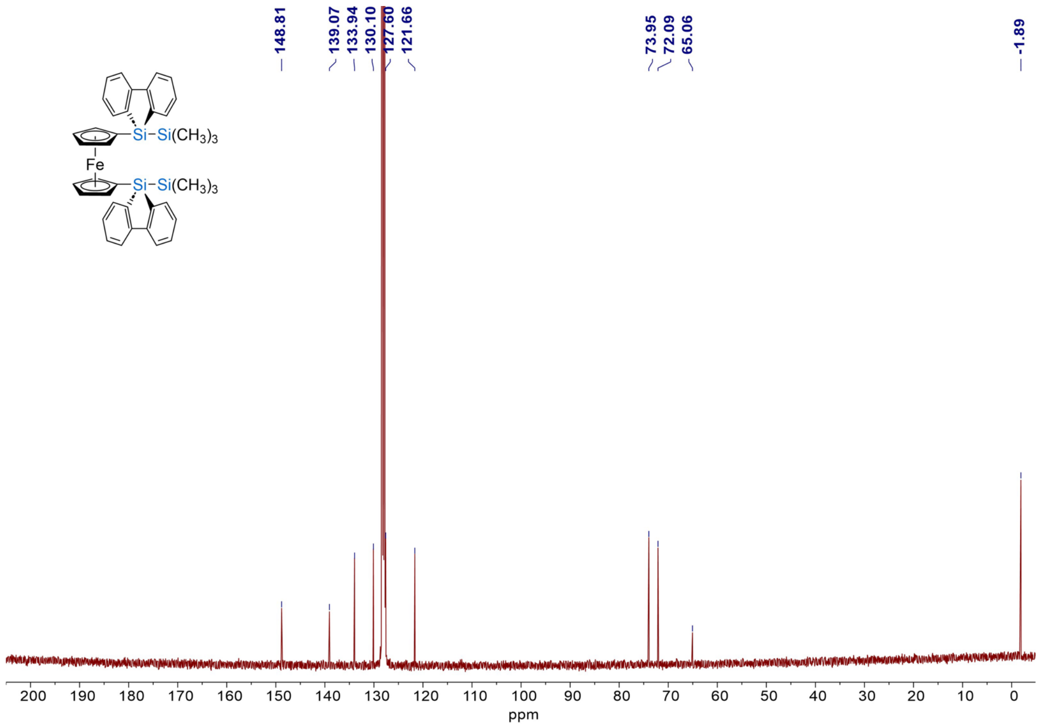

Figure S13. $^{13}C\{^{1}H\}$ NMR spectrum of **6** (C_6D_6, 101 MHz).

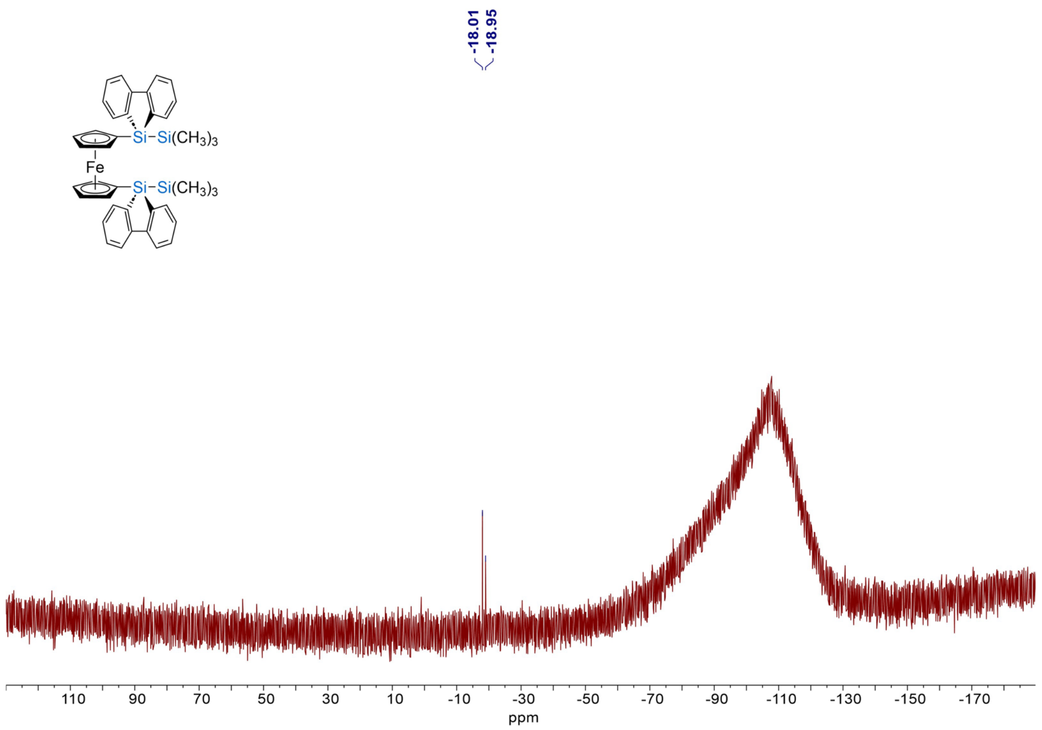

Figure S14. $^{29}Si\{^{1}H\}$ NMR spectrum of **6** (C_6D_6, 79.5 MHz).

*1,1’-Bis(9-butyl-9-silafluorenyl)ferrocene (**8a**)*

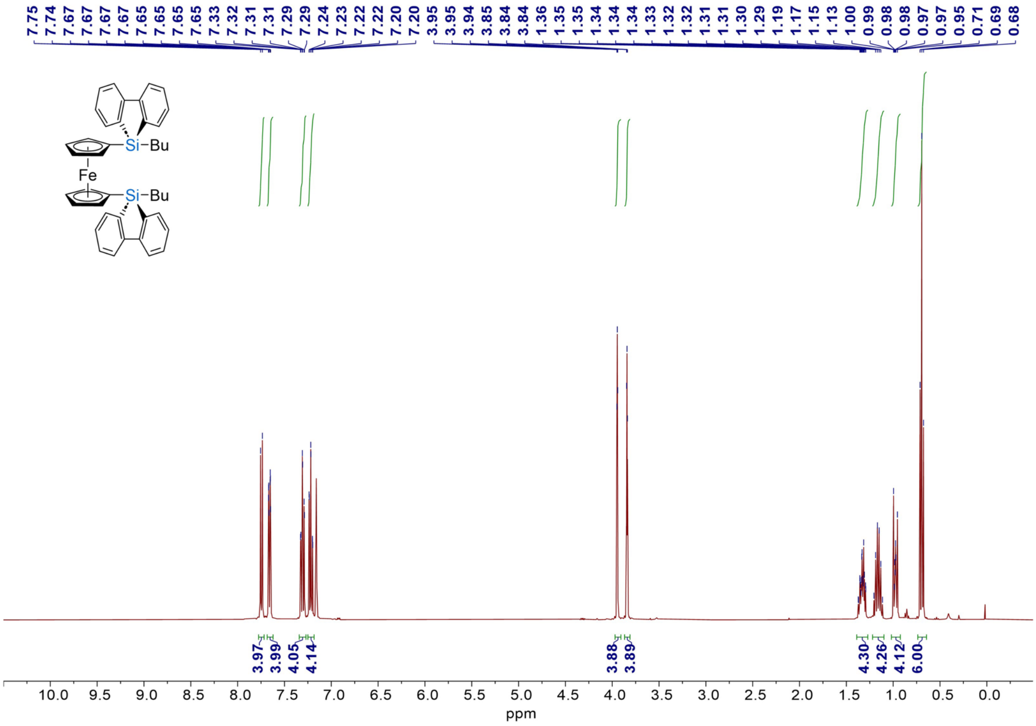

Figure S15. ^{1}H NMR spectrum of **8a** (C_6D_6, 400 MHz).

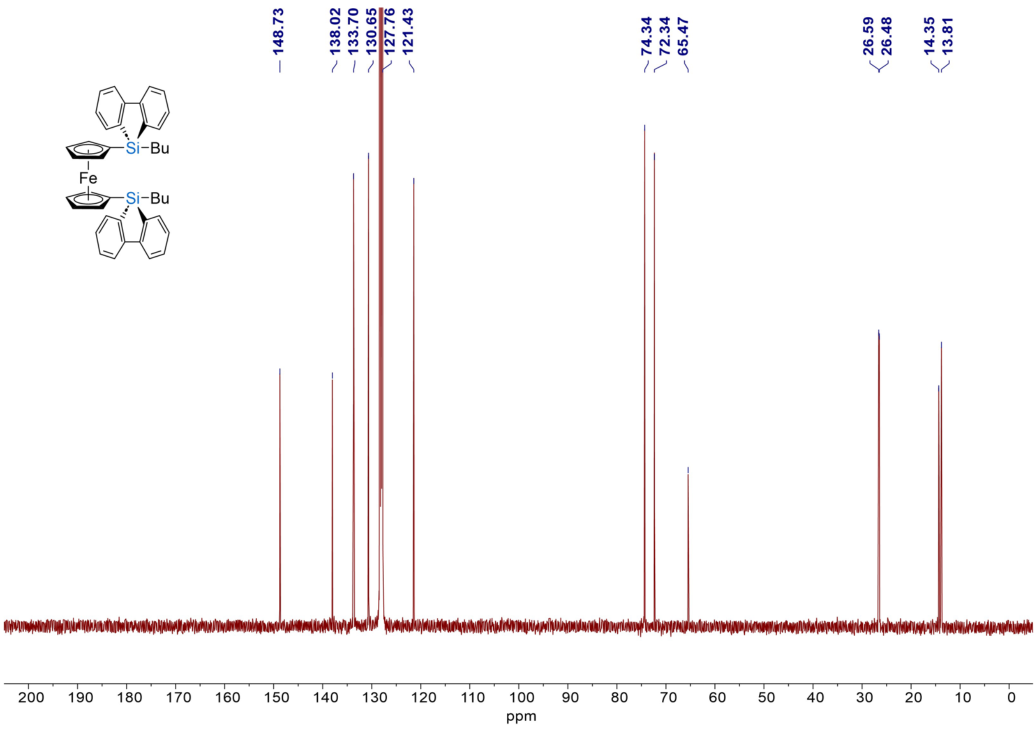

Figure S16. $^{13}C\{^{1}H\}$ NMR spectrum of **8a** (C_6D_6, 101 MHz).

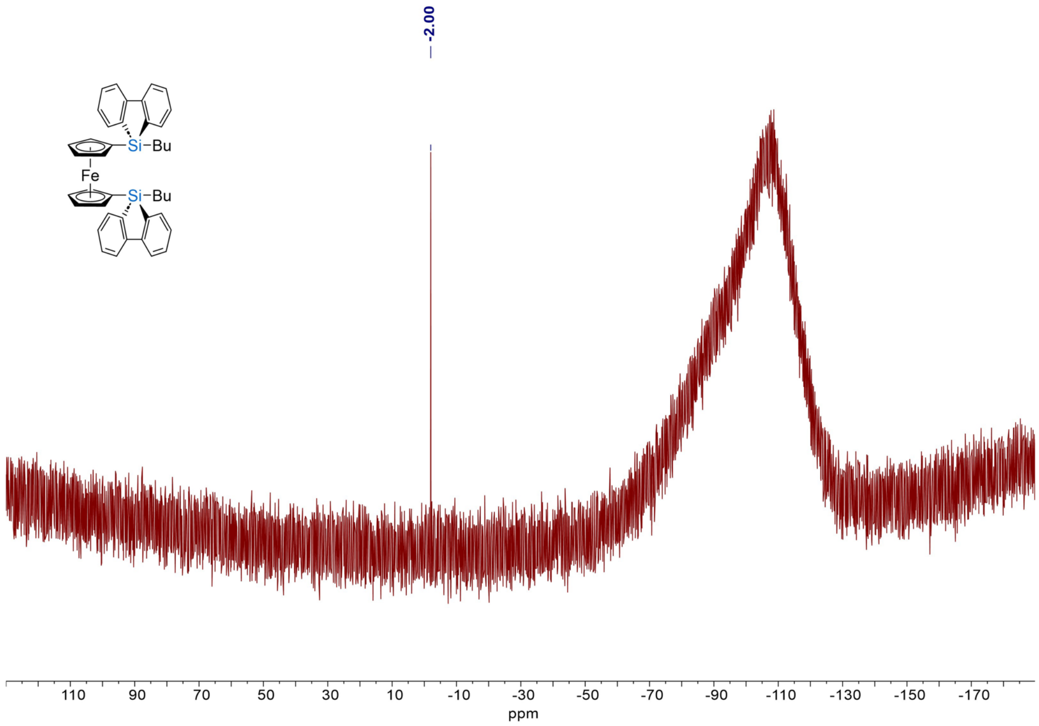

Figure S17. $^{29}Si\{^1H\}$ NMR spectrum of **8a** (C_6D_6, 79.5 MHz).

*1,1'-Bis(9-chlorobutyl-9-silafluorenyl)ferrocene (**8b**)*

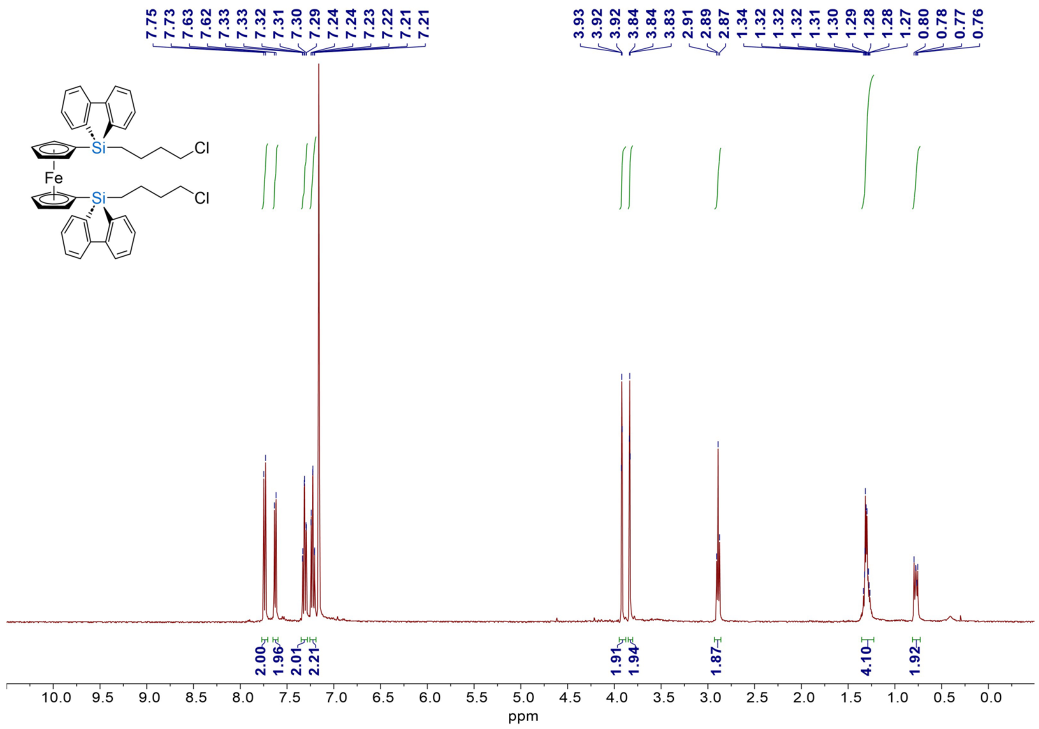

Figure S18. ^1H NMR spectrum of **8b** (C_6D_6, 400 MHz).

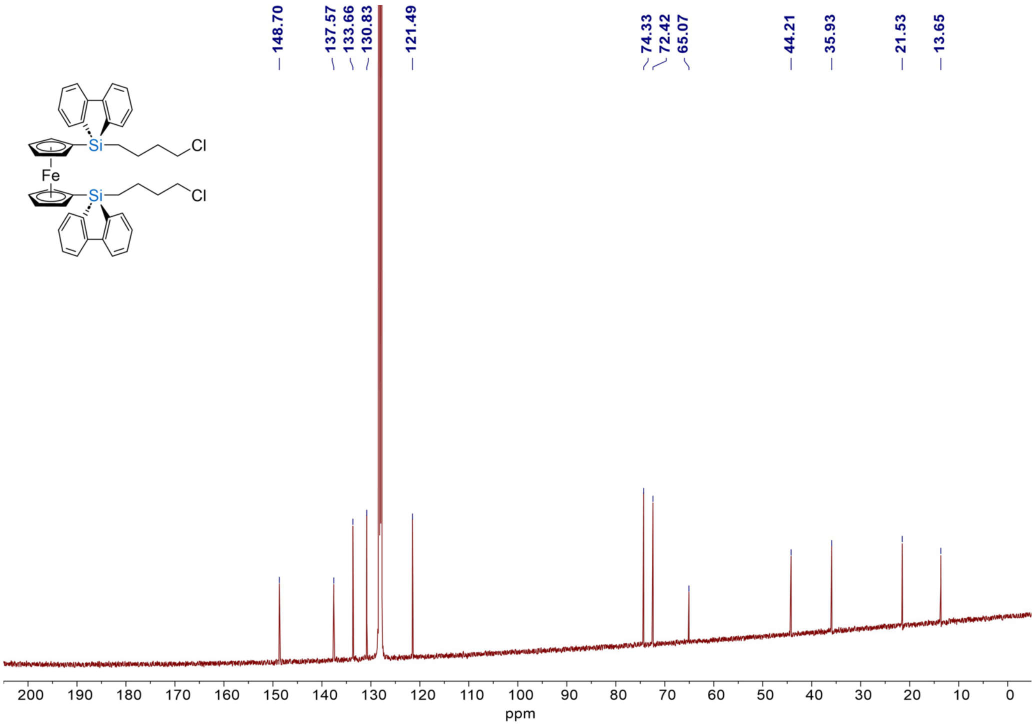

Figure S19. $^{13}C\{^{1}H\}$ NMR spectrum of **8b** (C_6D_6, 101 MHz).

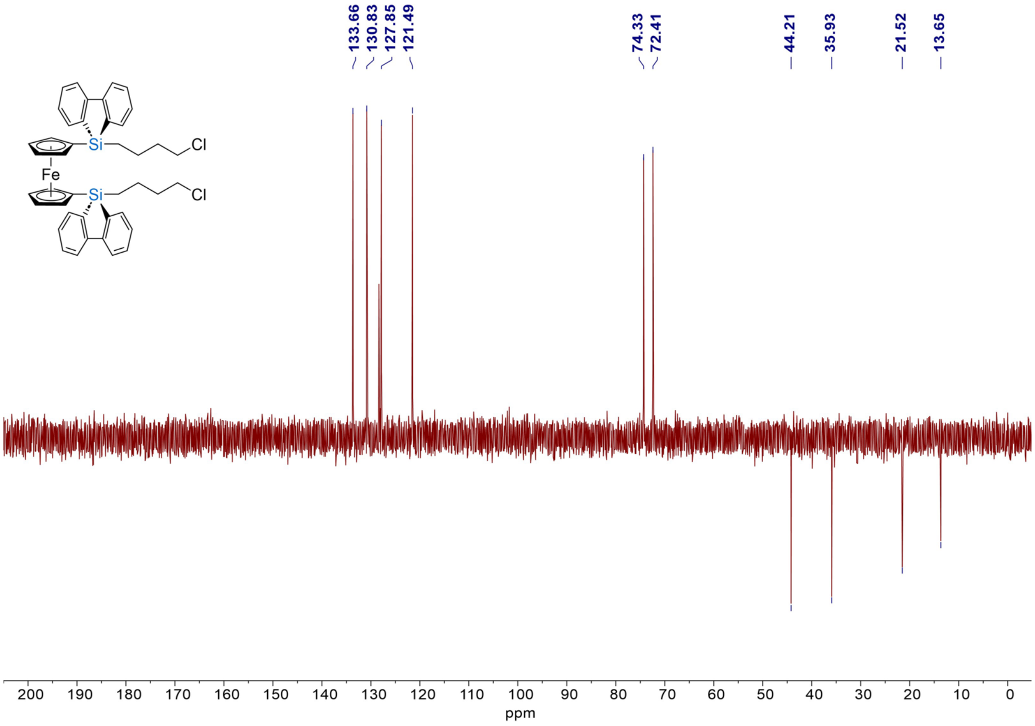

Figure S20. DEPT135 $^{13}C\{^{1}H\}$ NMR spectrum of **8b** (C_6D_6, 101 MHz).

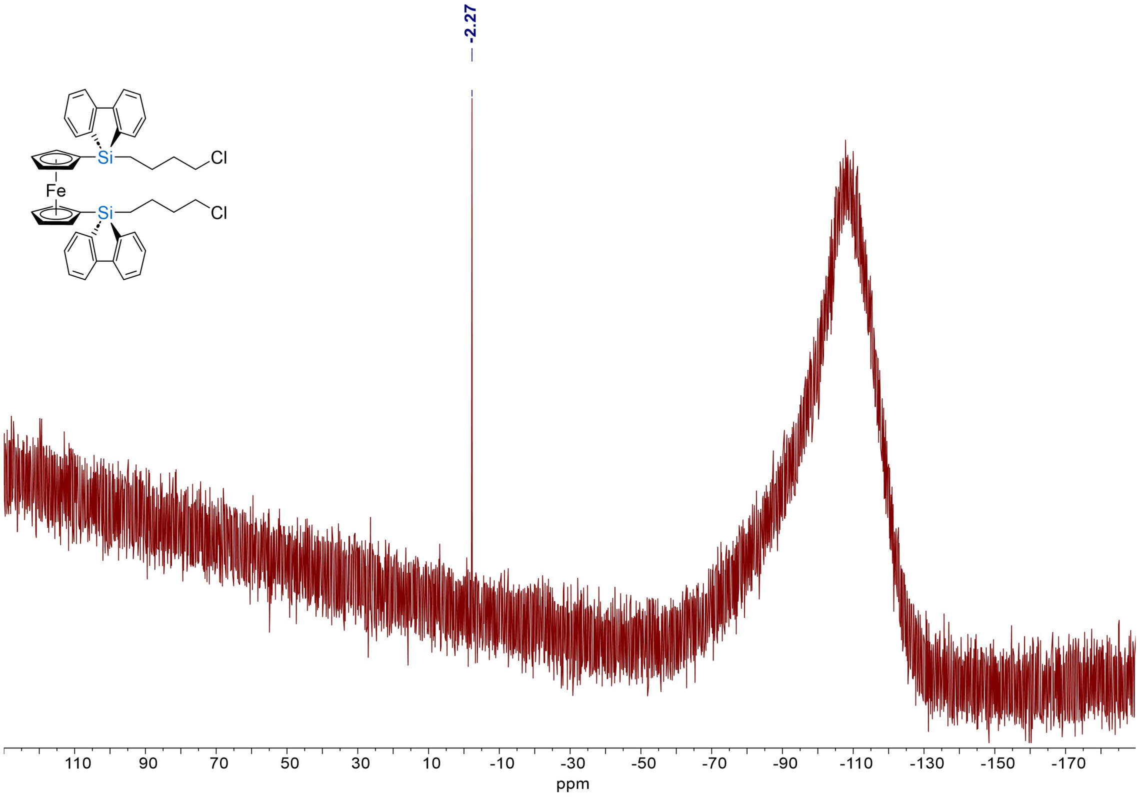

Figure S21. $^{29}Si\{^1H\}$ NMR spectrum of **8b** (C_6D_6, 79.5 MHz).

Trisila[3]ferrocenophane ***9***

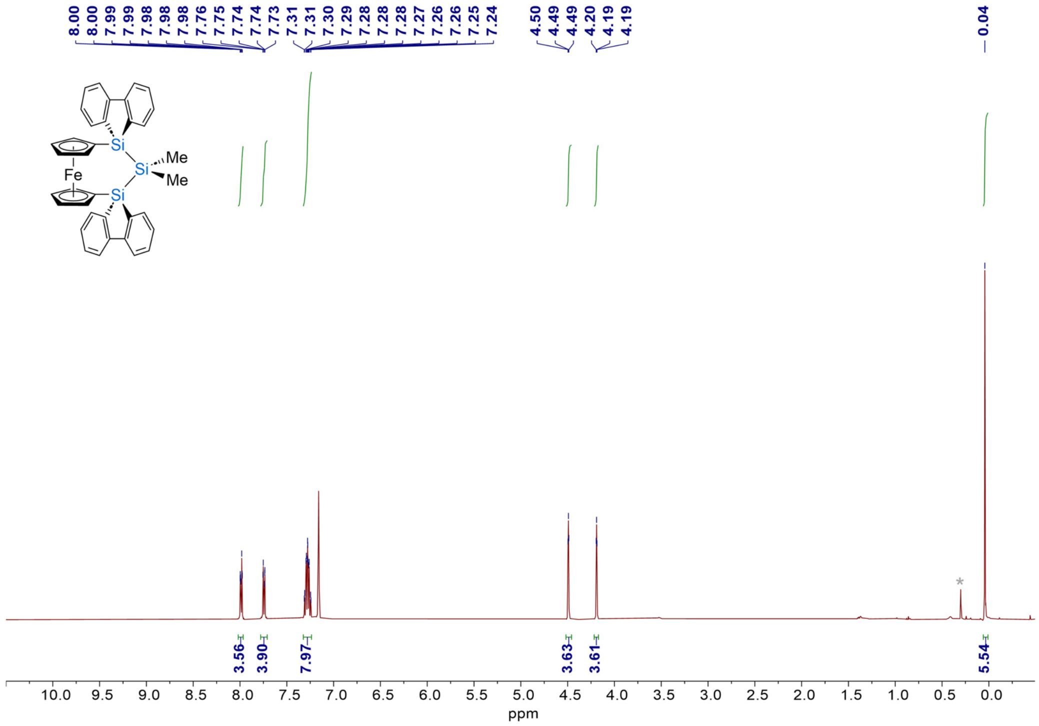

Figure S22. ^1H NMR spectrum of **9** (C_6D_6, 400 MHz, *: silicon grease).

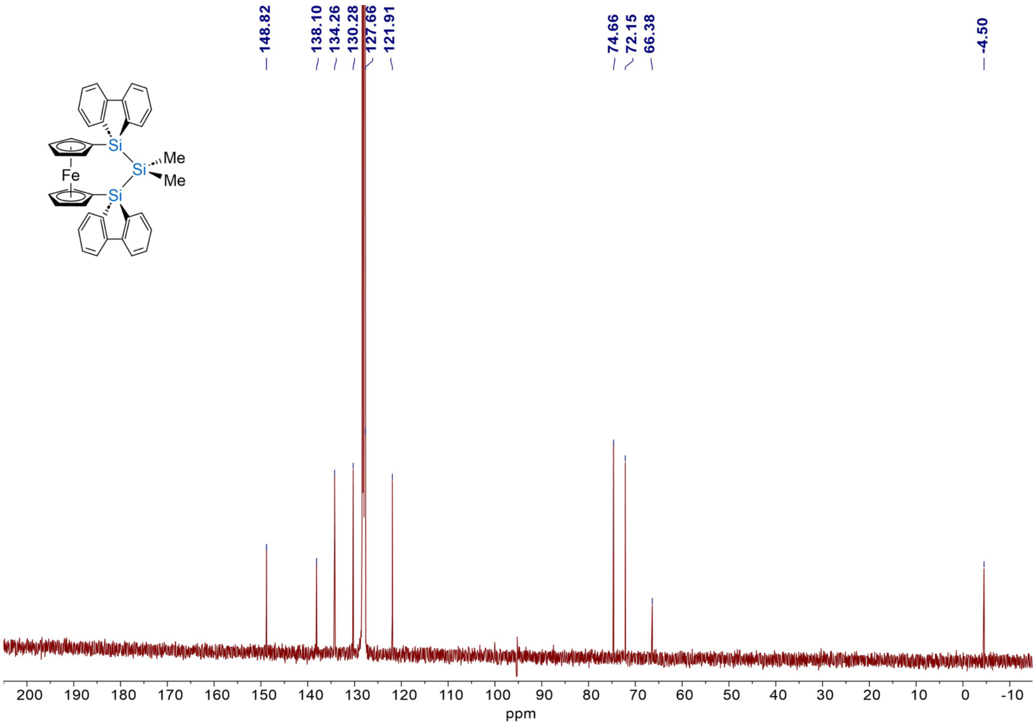

Figure S23. $^{13}C\{^{1}H\}$ NMR spectrum of **9** (C_6D_6, 101 MHz).

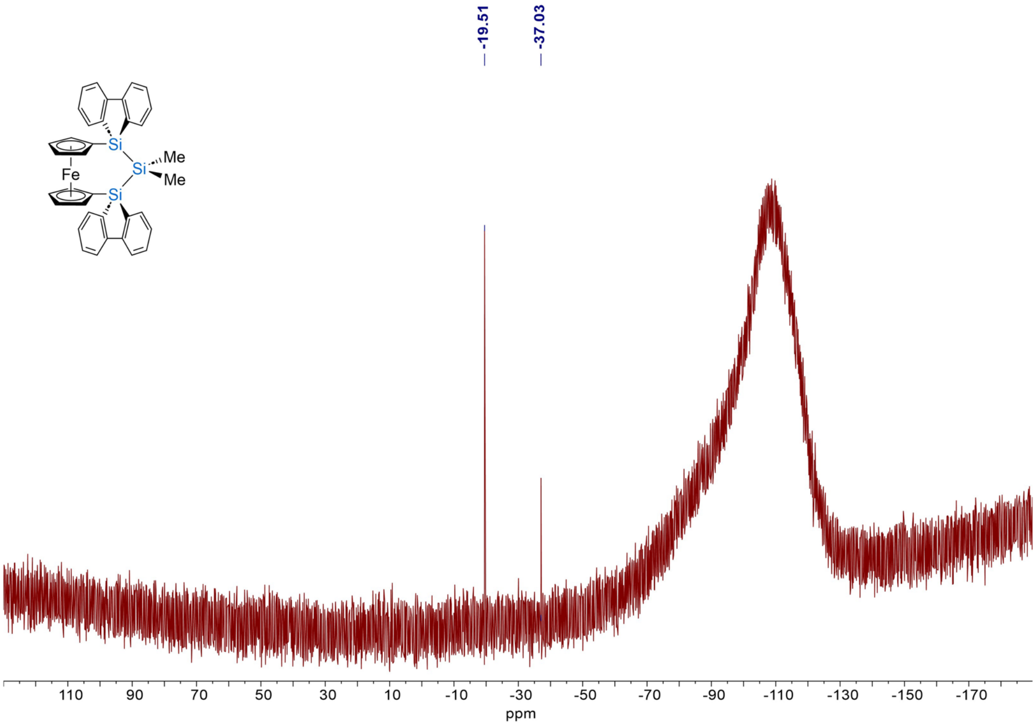

Figure S24. $^{29}Si\{^{1}H\}$ NMR spectrum of **9** (C_6D_6, 79.5 MHz).

Trisila[3]ferrocenophane ***10***

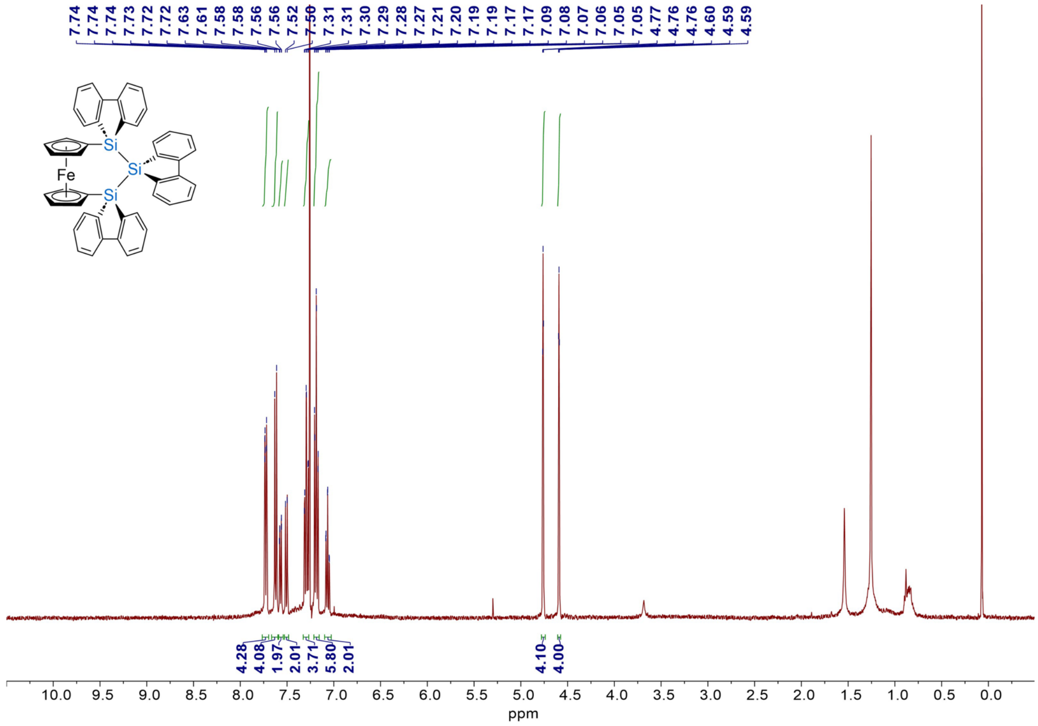

Figure S25. ^{1}H NMR spectrum of **10** (C_6D_6, 400 MHz).

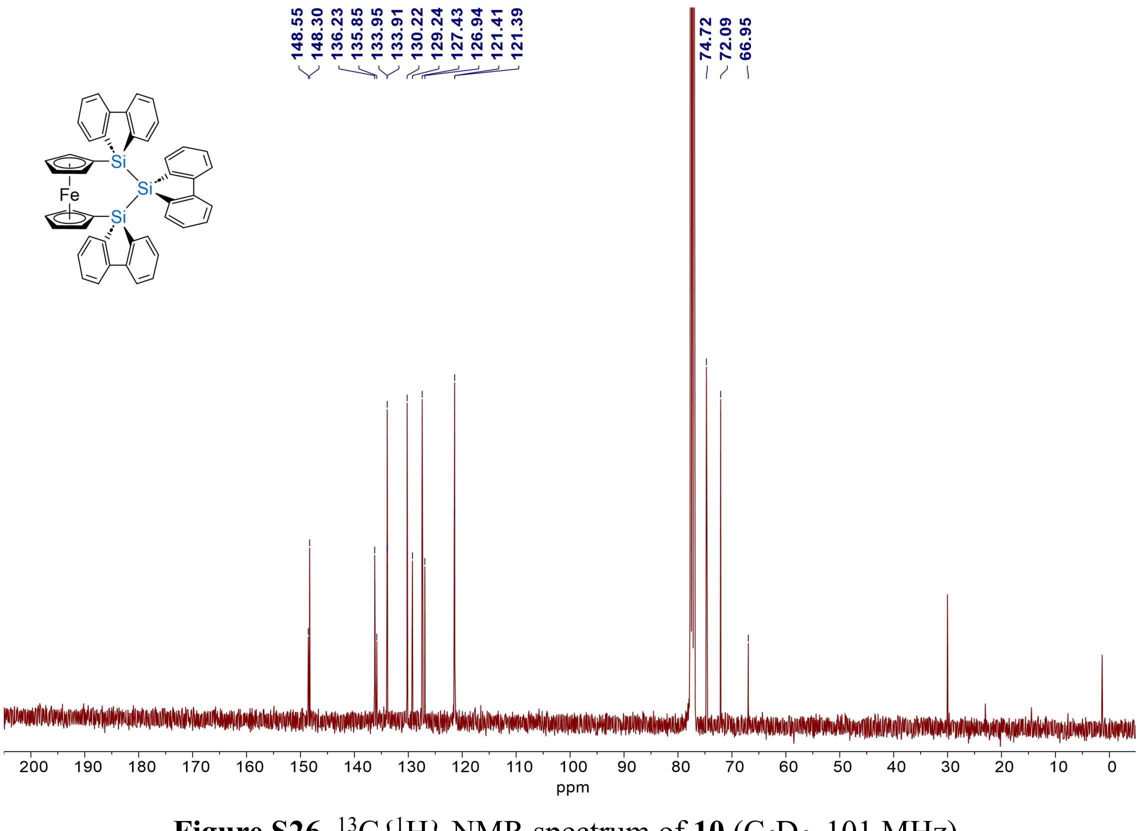

Figure S26. $^{13}C\{^{1}H\}$ NMR spectrum of **10** (C_6D_6, 101 MHz).

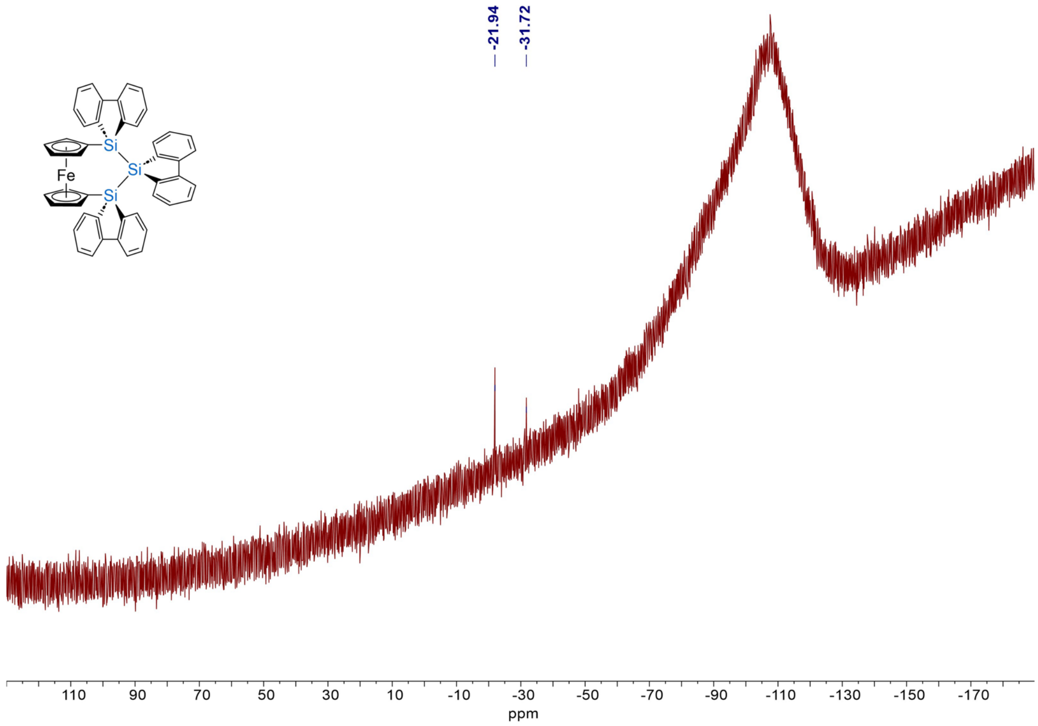

Figure S27. $^{29}Si\{^1H\}$ NMR spectrum of **10** (C_6D_6, 79.5 MHz).

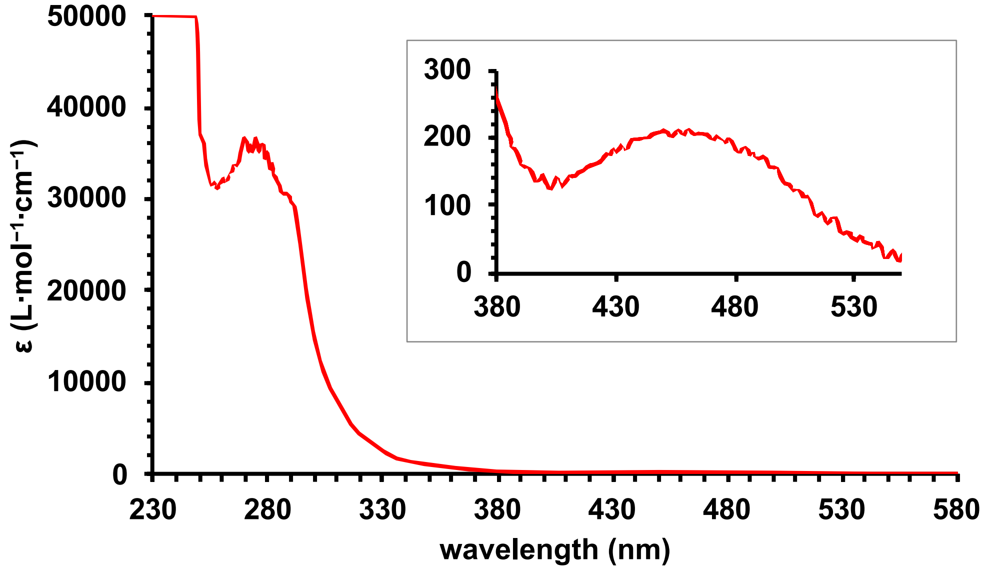

Figure S28. UV-Vis spectrum of **10** in THF at room temperature (0.1 mM).

2. Cartesian coordinates of theoretically optimized structures

Disila[2]ferrocenophane $\mathbf{1}_{opt}$

Fe	3.18945	0.00002	-0.00001
C	1.96685	-0.36000	1.61819
C	3.01587	-1.33145	1.55288
H	2.87640	-2.39570	1.42897
C	4.26687	-0.66141	1.62058
H	5.24041	-1.12720	1.56064
C	4.01224	0.73184	1.73116
H	4.75750	1.51336	1.77554
C	2.60389	0.92007	1.72304
H	2.09254	1.87130	1.75976
C	1.96682	0.36006	-1.61818
C	2.60386	-0.92000	-1.72307
H	2.09252	-1.87124	-1.75981
C	4.01221	-0.73176	-1.73121
H	4.75748	-1.51327	-1.77564
C	4.26683	0.66148	-1.62060
H	5.24037	1.12728	-1.56067
C	3.01582	1.33151	-1.55286
H	2.87635	2.39576	-1.42892
Si	0.24374	-0.61315	0.97840
C	-0.30795	-2.34320	0.57556
C	0.34098	-3.42296	-0.01301
H	1.41033	-3.38176	-0.19790
C	-0.36775	-4.56502	-0.36616
H	0.14580	-5.40857	-0.81718
C	-1.73979	-4.62454	-0.14101
H	-2.29448	-5.51547	-0.41933
C	-2.40493	-3.55480	0.44055
H	-3.47538	-3.61847	0.60759
C	-1.70151	-2.40915	0.80630
C	-1.38686	-0.17851	1.75565
C	-1.86239	1.03101	2.24795
H	-1.17260	1.83919	2.47247
C	-3.22593	1.22698	2.42706
H	-3.59213	2.17216	2.81529
C	-4.12251	0.22045	2.08418
H	-5.18913	0.38257	2.20677
C	-3.66708	-0.98496	1.56869
H	-4.38350	-1.74612	1.27720
C	-2.30126	-1.19949	1.40412
Si	0.24370	0.61316	-0.97836
C	-1.38688	0.17847	-1.75561
C	-1.86238	-1.03107	-2.24789
H	-1.17257	-1.83924	-2.47239
C	-3.22591	-1.22707	-2.42703
H	-3.59208	-2.17226	-2.81525
C	-4.12252	-0.22055	-2.08419
H	-5.18913	-0.38270	-2.20679
C	-3.66712	0.98487	-1.56870
H	-4.38356	1.74602	-1.27725
C	-2.30131	1.19944	-1.40411
C	-0.30804	2.34320	-0.57555
C	0.34086	3.42300	0.01301
H	1.41021	3.38183	0.19791
C	-0.36790	4.56505	0.36613
H	0.14562	5.40862	0.81714
C	-1.73994	4.62453	0.14097
H	-2.29465	5.51545	0.41927
C	-2.40505	3.55476	-0.44057
H	-3.47550	3.61839	-0.60762
C	-1.70159	2.40912	-0.80630

Disila[2]ferrocenophane dimer $\mathbf{1}_{2opt}$

H	3.8145	2.9370	4.9221
H	4.9639	-2.0505	-2.4111
H	-1.5131	-1.6586	-2.8610
H	6.5833	0.6860	-2.1657
H	1.7254	-1.8240	4.9134
C	0.7164	-1.4630	-0.3236
H	5.3529	-2.7302	1.8705
C	6.6580	-3.3419	0.1645
H	7.4813	-3.8397	0.6571
Fe	6.5627	-1.3358	-0.2328
C	6.2140	0.6563	0.0429
C	0.5748	-1.2094	-3.0949
C	-0.4939	-1.6436	-0.9811
Si	4.4123	1.1032	0.1828
H	0.5094	-1.1033	-4.1728
C	3.0896	4.2095	3.3480
C	-0.5622	-1.5237	-2.3612
C	7.0241	0.1288	1.0995
C	3.8011	2.3504	-1.0637
C	3.9933	2.2363	1.6043
C	3.1698	3.3547	-3.1640
C	0.2128	-1.9991	3.3953
H	2.1697	5.2511	-3.0384
C	3.2734	3.4665	-0.3759
C	4.6938	-2.1591	-0.1903
H	-1.3874	-1.8751	-0.4165
C	3.7379	2.3052	-2.4508
H	2.7307	4.9737	4.0303
C	5.5319	-2.7544	0.8046
H	4.1286	1.4453	-2.9874
H	3.1301	3.3201	-4.2483
C	-0.0887	-1.9144	2.0432
Si	3.2990	-0.9581	0.1191
H	6.7585	0.1090	2.1473

C	8.2159	-0.4076	0.5384
H	9.0089	-0.9000	1.0833
C	8.1585	-0.2206	-0.8694
C	2.6815	4.5009	-1.0966
C	2.5099	-1.4418	2.9456
C	2.6353	4.4430	-2.4833
H	8.9001	-0.5460	-1.5852
C	2.2297	-1.3661	1.5888
C	6.9324	0.4319	-1.1747
H	3.5166	-1.2535	3.3096
H	2.2501	5.3545	-0.5839
H	-1.1060	-2.0801	1.7113
C	4.1393	2.0770	2.9764
H	-0.5763	-2.2423	4.0994
H	4.5932	1.1753	3.3771
C	3.6977	3.0642	3.8504
C	1.5036	-1.7614	3.8522
C	0.9116	-1.5945	1.1329
C	6.5305	-3.1199	-1.2338
C	1.8714	-1.1278	-1.0636
C	1.7859	-1.0010	-2.4438
H	7.2391	-3.4197	-1.9930
C	5.3261	-2.3952	-1.4525
H	2.6637	-0.7322	-3.0252
C	2.9309	4.3827	1.9796
H	2.4467	5.2800	1.6080
C	3.3872	3.4068	1.0978
C	-1.8714	1.1278	1.0636
C	-0.7164	1.4630	0.3236
H	-1.7254	1.8240	-4.9134
C	-0.2128	1.9991	-3.3953
H	-4.9639	2.0505	2.4111
C	-0.5748	1.2094	3.0949
H	-0.5094	1.1033	4.1728
C	0.5622	1.5237	2.3612
C	0.0887	1.9144	-2.0432
H	1.5131	1.6586	2.8610
H	-2.1697	-5.2511	3.0384
Si	-4.4123	-1.1032	-0.1828
C	-1.7859	1.0010	2.4438
C	-2.9309	-4.3827	-1.9796
C	-6.5305	3.1199	1.2338
H	-2.4467	-5.2800	-1.6080
C	-2.5099	1.4418	-2.9456
H	-3.5166	1.2535	-3.3096
C	-8.1585	0.2206	0.8694
H	-2.6637	0.7322	3.0252
H	1.1060	2.0801	-1.7113
C	0.4939	1.6436	0.9811
H	-7.2391	3.4197	1.9930
H	-6.5833	-0.6860	2.1657
C	-3.1698	-3.3547	3.1640
C	-3.2734	-3.4665	0.3759
C	-1.5036	1.7614	-3.8522
C	-4.6938	2.1591	0.1903
C	-5.3261	2.3952	1.4525
C	-0.9116	1.5945	-1.1329
H	1.3874	1.8751	0.4165
C	-5.5319	2.7544	-0.8046
H	-3.1301	-3.3201	4.2483
H	-5.3529	2.7302	-1.8705
Fe	-6.5627	1.3358	0.2328
C	-6.6580	3.3419	-0.1645
Si	-3.2990	0.9581	-0.1191
C	-6.2140	-0.6563	-0.0429
H	0.5763	2.2423	-4.0994
C	-2.6815	-4.5009	1.0966
H	-7.4813	3.8397	-0.6571
H	-8.9001	0.5460	1.5852
H	-2.2501	-5.3545	0.5839
C	-3.9933	-2.2363	-1.6043
C	-7.0241	-0.1288	-1.0995
C	-2.2297	1.3661	-1.5888
C	-2.6353	-4.4430	2.4833
C	-6.9324	-0.4319	1.1747
C	-3.8011	-2.3504	1.0637
C	-3.7379	-2.3052	2.4508
H	-3.8145	-2.9370	-4.9221
H	-4.1286	-1.4453	2.9874
C	-3.0896	-4.2095	-3.3480
H	-2.7307	-4.9737	-4.0303
H	-6.7585	-0.1090	-2.1473
C	-8.2159	0.4076	-0.5384
C	-4.1393	-2.0770	-2.9764
H	-9.0089	0.9000	-1.0833
H	-4.5932	-1.1753	-3.3771
C	-3.6977	-3.0642	-3.8504
C	-3.3872	-3.4068	-1.0978

*1,1'-Bis(9-trimethylsilyl-9-silafluorenyl)ferrocene (**6**$_{opt}$)*

Fe	0.0000	0.0000	-0.1391
C	1.6182	-1.2441	-0.0895
C	0.8402	-1.4736	-1.2700
H	1.1793	-1.3215	-2.2853
C	-0.4725	-1.8535	-0.8866
H	-1.3017	-2.0421	-1.5522
C	-0.5257	-1.8675	0.5339
H	-1.4007	-2.0673	1.1336
C	0.7530	-1.4951	1.0237
H	1.0138	-1.3599	2.0641
Si	3.3621	-0.6398	-0.0226
C	3.6982	0.7171	-1.2626
C	3.6483	0.7200	-2.6506
H	3.4115	-0.1927	-3.1907
C	3.8874	1.8912	-3.3617
H	3.8374	1.8921	-4.4463
C	4.1903	3.0648	-2.6791
H	4.3747	3.9798	-3.2336
C	4.2596	3.0759	-1.2922
H	4.4962	4.0021	-0.7778
C	4.0148	1.9079	-0.5733
C	3.6772	0.5232	1.4058
C	3.6244	0.3224	2.7791
H	3.3683	-0.6553	3.1782
C	3.8912	1.3692	3.6555
H	3.8402	1.2100	4.7283
C	4.2263	2.6222	3.1541
H	4.4373	3.4389	3.8377
C	4.2944	2.8382	1.7838
H	4.5578	3.8235	1.4117
C	4.0187	1.7972	0.9001
Si	4.9470	-2.3416	-0.1233
C	4.7278	-3.2848	-1.7339
H	4.8844	-2.6288	-2.5949
H	3.7201	-3.7023	-1.8102
H	5.4403	-4.1119	-1.8133
C	4.6957	-3.4914	1.3414
H	3.6855	-3.9098	1.3430
H	5.4043	-4.3254	1.3221
H	4.8380	-2.9575	2.2852
C	6.6425	-1.5404	-0.0521
H	6.7779	-0.8475	-0.8869
H	6.7610	-0.9686	0.8722
H	7.4453	-2.2830	-0.0954
C	-1.6180	1.2442	-0.0892
C	-0.7529	1.4949	1.0241
H	-1.0138	1.3594	2.0645
C	0.5259	1.8674	0.5343
H	1.4008	2.0668	1.1342
C	0.4727	1.8537	-0.8861
H	1.3018	2.0423	-1.5517
C	-0.8401	1.4740	-1.2696
H	-1.1792	1.3221	-2.2850
Si	-3.3619	0.6396	-0.0225
C	-3.6771	-0.5235	1.4059
C	-3.6244	-0.3228	2.7791
H	-3.3680	0.6548	3.1784
C	-3.8915	-1.3695	3.6555
H	-3.8406	-1.2104	4.7282
C	-4.2270	-2.6224	3.1540
H	-4.4383	-3.4392	3.8374
C	-4.2950	-2.8383	1.7836
H	-4.5586	-3.8235	1.4116
C	-4.0190	-1.7973	0.9000
C	-3.6981	-0.7171	-1.2626
C	-3.6484	-0.7199	-2.6506
H	-3.4113	0.1927	-3.1906
C	-3.8880	-1.8910	-3.3618
H	-3.8381	-1.8916	-4.4463
C	-4.1913	-3.0645	-2.6793
H	-4.3761	-3.9795	-3.2338
C	-4.2604	-3.0757	-1.2924
H	-4.4973	-4.0019	-0.7781
C	-4.0151	-1.9079	-0.5733
Si	-4.9466	2.3415	-0.1232
C	-4.6948	3.4912	1.3416
H	-4.8374	2.9573	2.2854
H	-3.6845	3.9091	1.3432
H	-5.4030	4.3255	1.3223
C	-4.7267	3.2847	-1.7337
H	-3.7188	3.7018	-1.8098
H	-5.4388	4.1122	-1.8131
H	-4.8835	2.6289	-2.5948
C	-6.6425	1.5413	-0.0522
H	-6.7615	0.9696	0.8721
H	-6.7782	0.8485	-0.8870
H	-7.4449	2.2843	-0.0957
